# Supplementary figures and images for: Testing the mutant selection window hypothesis with meropenem: In vitro model study with OXA-48-producing Klebsiella pneumoniae
Source: PLoS One. 2023 Aug 4;18(8):e0288660. doi: 10.1371/journal.pone.0288660 (PMC10403107; doi:10.1371/journal.pone.0288660)

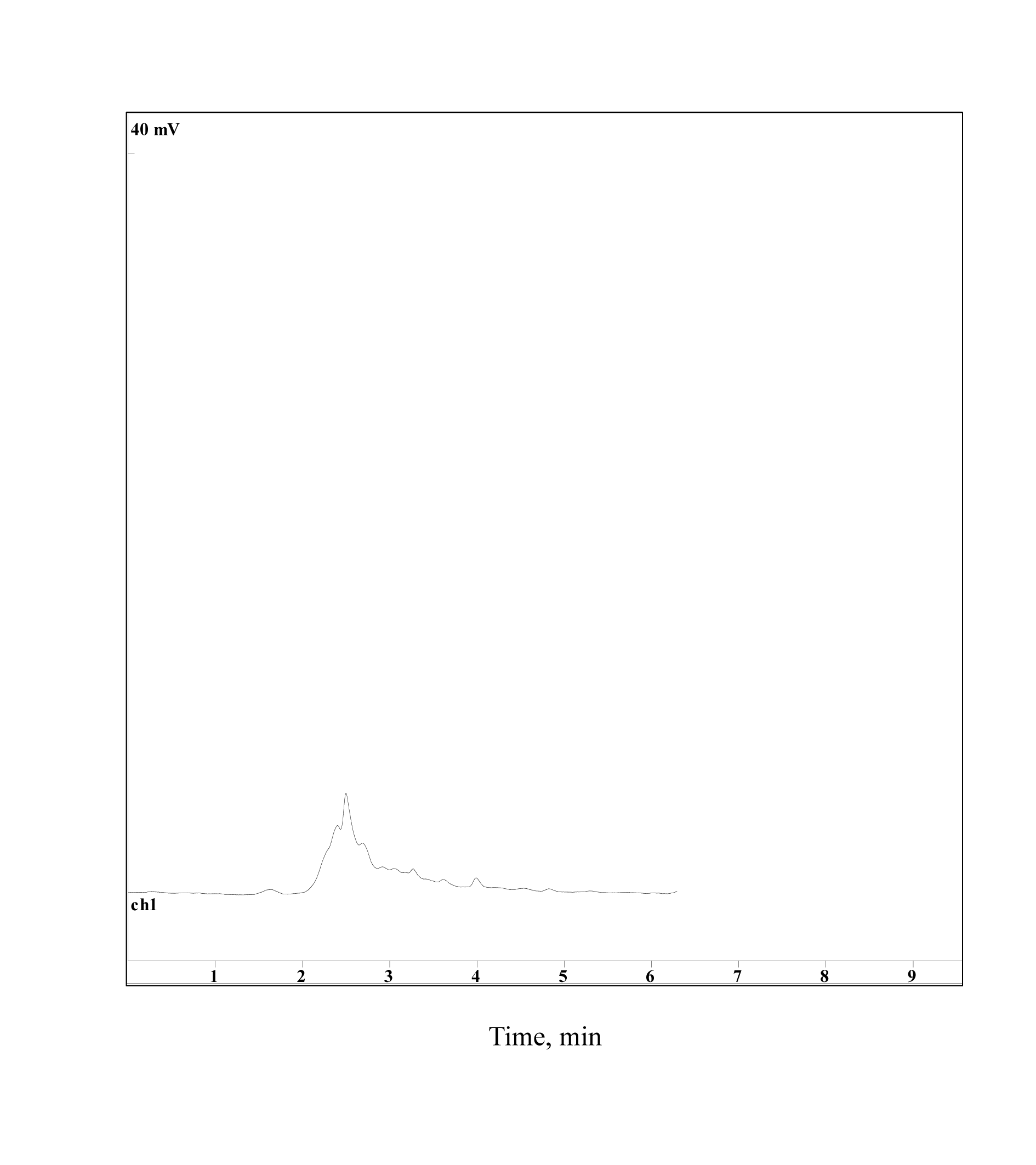

Supplement: S1 Fig — (TIF) [file pone.0288660.s001.tif]

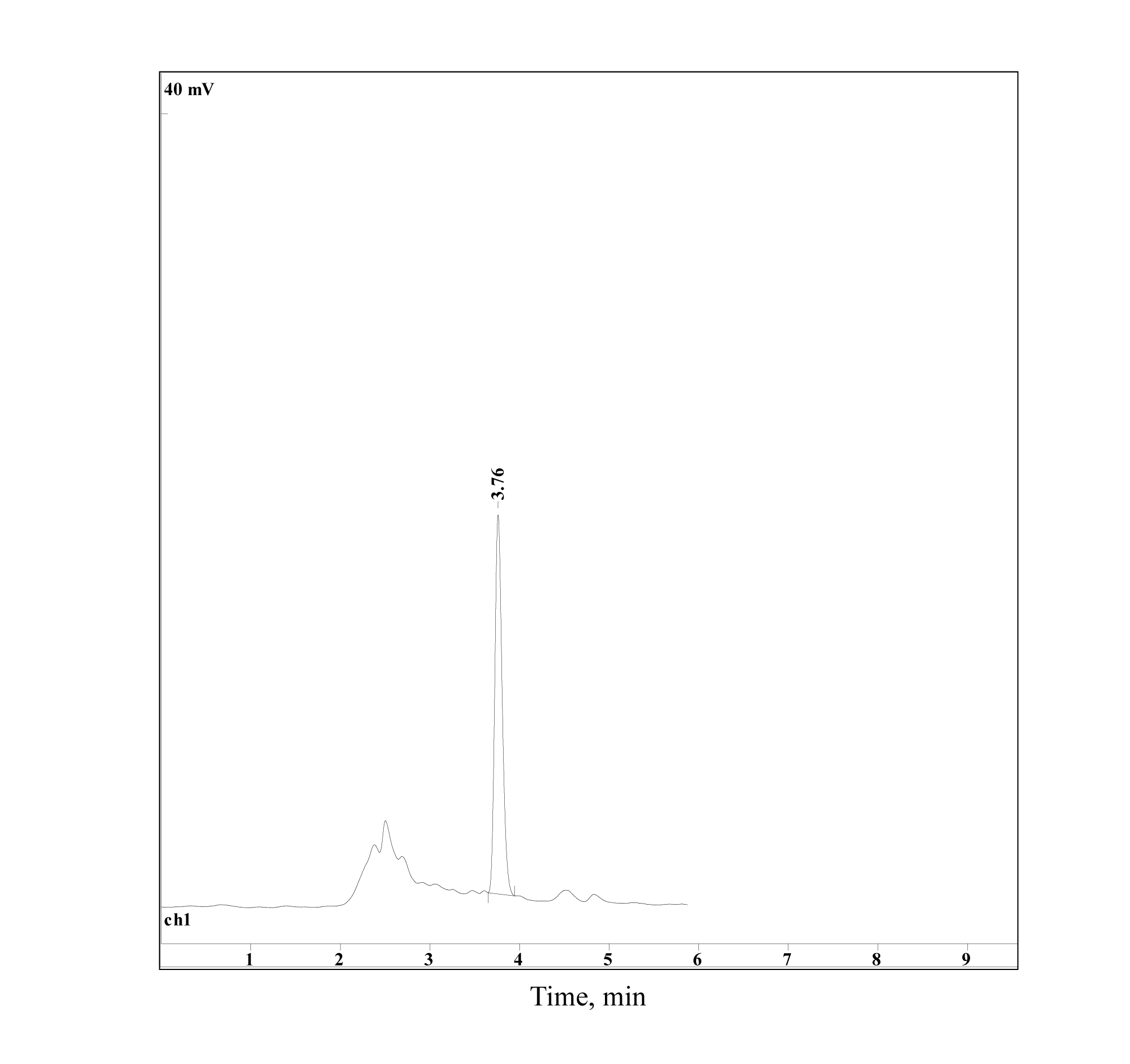

Supplement: S2 Fig — The meropenem peak shows a characteristic retention time of 3.76 minutes. (TIF) [file pone.0288660.s002.tif]

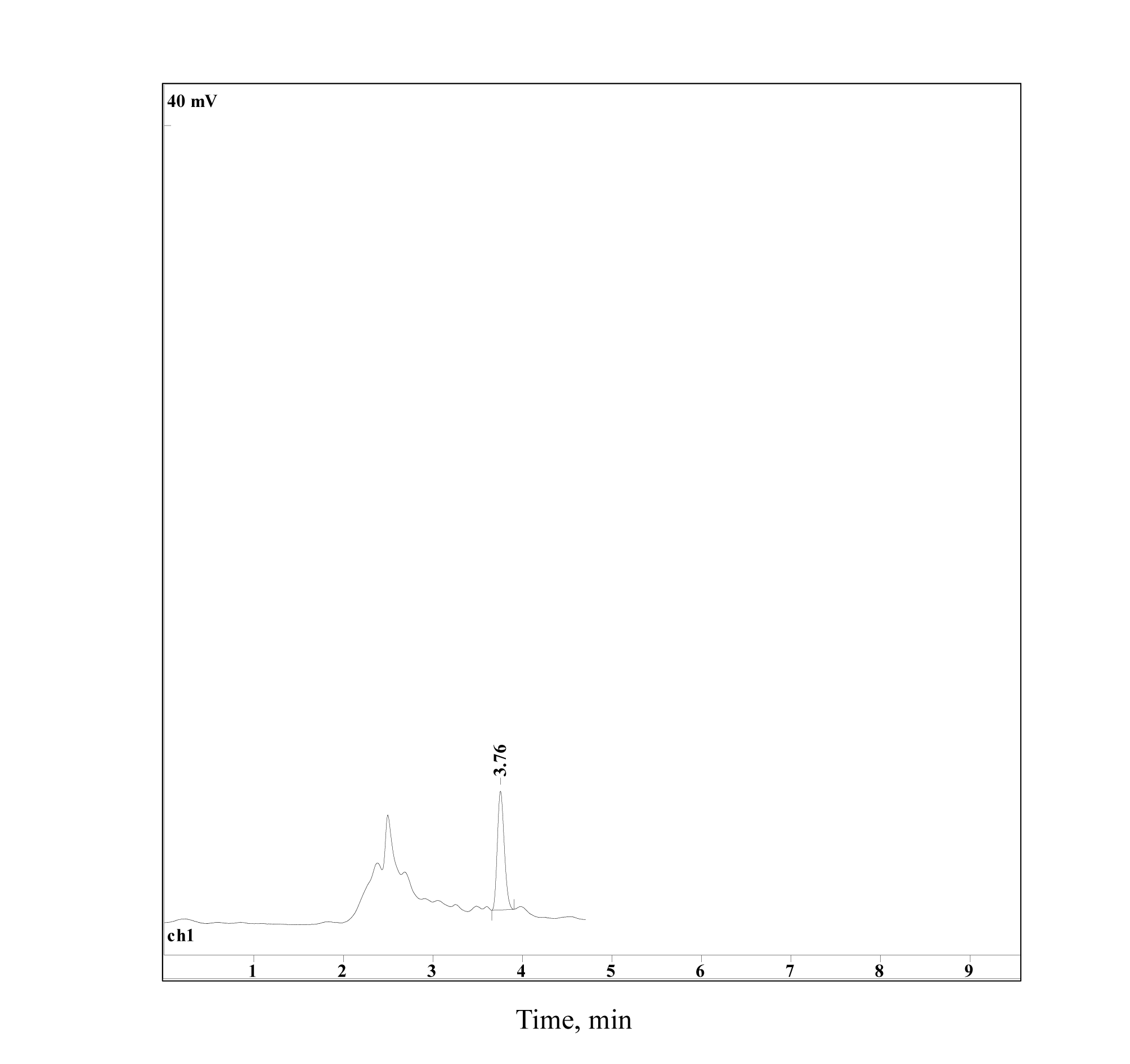

Supplement: S3 Fig — (TIF) [file pone.0288660.s003.tif]

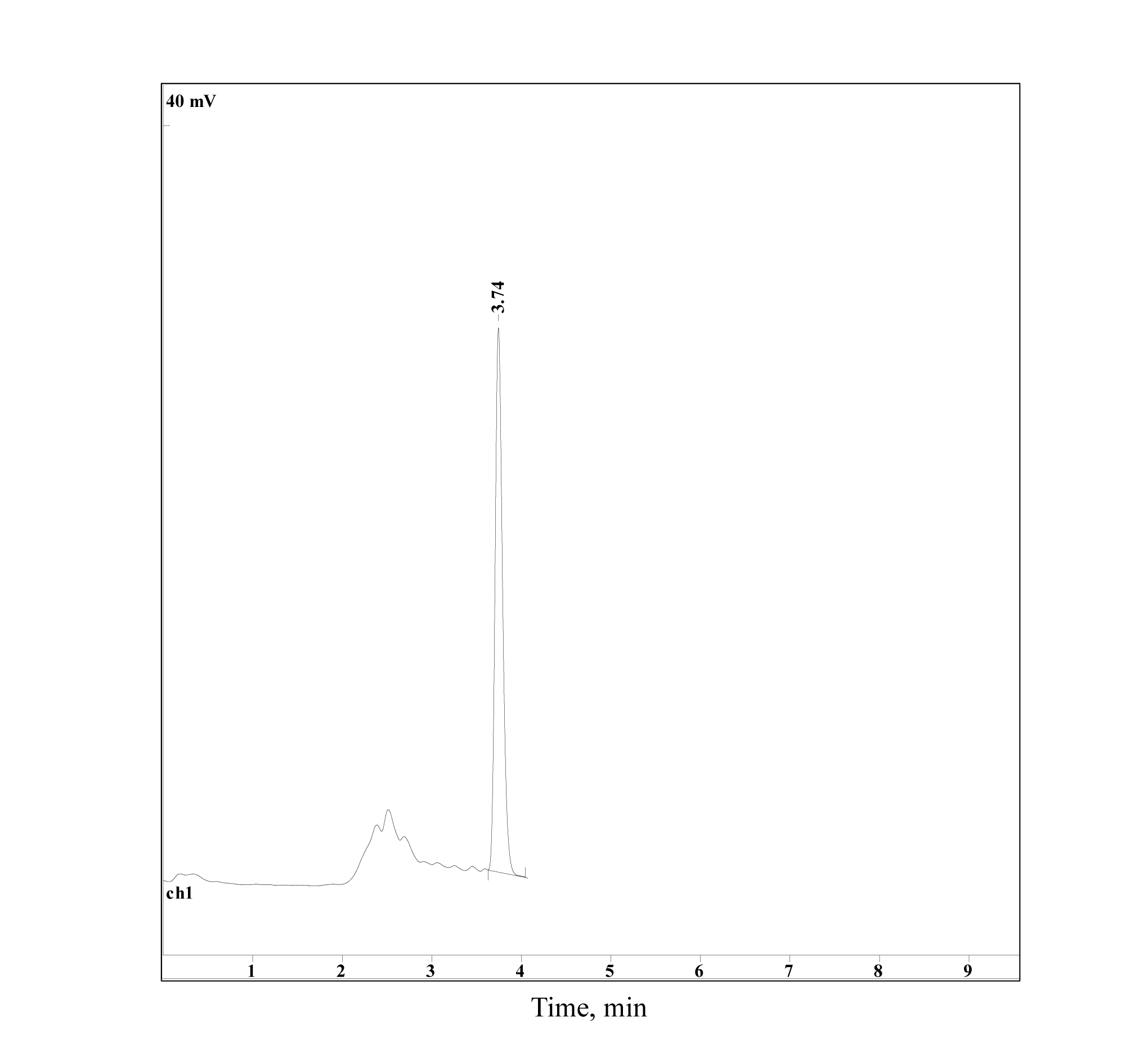

Supplement: S4 Fig — (TIF) [file pone.0288660.s004.tif]

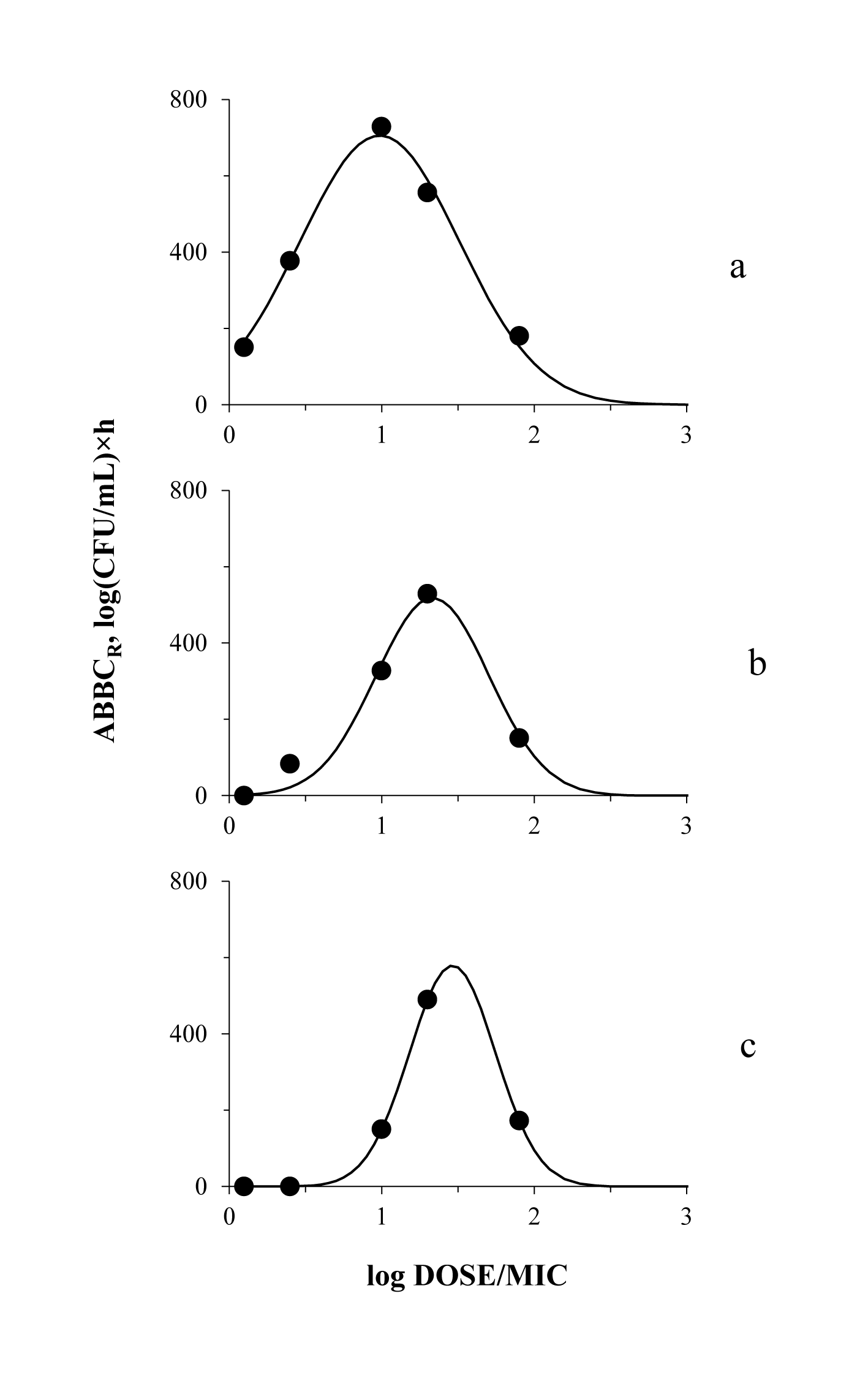

Supplement: S5 Fig — DOSE/MIC relationship with ABBCR for mutants resistant to 2×MIC (a), 8×MIC (b) and 16×MIC (c) of meropenem; data fitted by Eq (2). (TIF) [file pone.0288660.s005.tif]
